# Immunization with an Autotransporter Protein of *Orientia tsutsugamushi* Provides Protective Immunity against Scrub Typhus

**DOI:** 10.1371/journal.pntd.0003585

**Published:** 2015-03-13

**Authors:** Na-Young Ha, Prashant Sharma, Gwanghun Kim, Yuri Kim, Chan-Ki Min, Myung-Sik Choi, Ik-Sang Kim, Nam-Hyuk Cho

**Affiliations:** 1 Department of Microbiology and Immunology, Seoul National University College of Medicine, Seoul, Republic of Korea; 2 Department of Biomedical Sciences, Seoul National University College of Medicine, Seoul, Republic of Korea; 3 Institute of Endemic Disease, Seoul National University Medical Research Center and Bundang Hospital, Seoul, Republic of Korea; University of Texas Medical Branch, UNITED STATES

## Abstract

**Background:**

Scrub typhus is an acute febrile disease caused by *Orientia tsutsugamushi* infection. Recently, the rapid increase of scrub typhus incidence in several countries within the endemic region has become a serious public health issue. Despite the wide range of preventative approaches that have been attempted in the past 70 years, all have failed to develop an effective prophylactic vaccine. Currently, the selection of the proper antigens is one of the critical barriers to generating cross-protective immunity against antigenically-variable strains of *O*. *tsutsugamushi*.

**Methodology/Principal Findings:**

We examined the potential role of ScaA protein, an autotransporter protein of *O*. *tsutsugamushi*, in bacterial pathogenesis and evaluated the protective attributes of ScaA immunization in lethal *O*. *tsutsugamushi* infection in mice. Our findings demonstrate that ScaA functions as a bacterial adhesion factor, and anti-ScaA antibody significantly neutralizes bacterial infection of host cells. In addition, immunization with ScaA not only provides protective immunity against lethal challenges with the homologous strain, but also confers significant protection against heterologous strains when combined with TSA56, a major outer membrane protein of *O*. *tsutsugamushi*.

**Conclusions/Significance:**

Immunization of ScaA proteins provides protective immunity in mice when challenged with the homologous strain and significantly enhanced protective immunity against infection with heterologous strains. To our knowledge, this is the most promising result of scrub typhus vaccination trials against infection of heterologous strains in mouse models thus far.

## Author Summary

Scrub typhus, caused by *Orientia tsutsugamushi* infection, is one of the common febrile illness in the Asia-Pacific region, accounting for up to 20% of febrile hospital admissions in rural areas of southern Asia. It has been estimated that one billion people are at risk and one million cases occur annually in the Asian-Pacific region. Despite continuous efforts to develop a vaccine against scrub typhus during the last several decades, all approaches have failed to induce effective immunity. The main issue for the development of a scrub typhus vaccine is the selection of proper antigens that cover a broad range of antigenic strains and induce long-lasting immunity. Here, we examined the potential use of ScaA protein as a vaccine antigen. Our findings demonstrate that ScaA protein functions as a bacterial adhesion factor and an antibody against ScaA significantly inhibits bacterial infection into host cells. In addition, ScaA vaccination provides protective immunity against lethal challenges of the homologous strain, and also confers better protection against heterologous strains when combined with TSA56, the major outer membrane protein that was previously used as a potential vaccine antigen. These results indicate that ScaA proteins could be used as a novel vaccine target for scrub typhus.

## Introduction

Scrub typhus is an acute febrile illness caused by *Orientia tsutsugamushi* infection. The bacterium is an obligate intracellular pathogen maintained through transovarian transmission in trombiculid mites that serve as vectors for the disease [1]. Humans are accidental hosts when infected larval mites feed on tissue fluids for their development. Early clinical manifestations begin with an eschar at the site of mite feeding and regional lymphadenopathy, followed by fever, headache, myalgia, and rash. Due to the lack of specificity of its early clinical presentation and the unavailability of rapid and effective diagnostic tests in local clinics, delayed treatment with proper antibiotics, such as doxycycline or chloramphenicol, is common and often leads to acute respiratory distress, renal failure, meningoencephalitis, gastrointestinal bleeding, and multiple organ failures in patients [2,3]. Bacterial load and the time of antibiotic initiation are critical factors that affect disease severity [4]. Several studies also reported scrub typhus cases that were poorly responsive to antibiotics [5]. The mortality rate of scrub typhus in the pre-antibiotic era reached up to 40% [1,6].

The endemic region of scrub typhus is geographically confined to south-eastern Asia, extending from Russia Far East and Korea in the north, to northern Australia in the south, Afghanistan in the west, and Japan and the western Pacific islands in the east [7]. It has been estimated that more than a million cases occur annually within this endemic region [8] and scrub typhus accounts for up to 20% of febrile hospital admissions in rural areas of southern Asia [9–12]. In addition, the rapid increase of scrub typhus incidence in China [13] and South Korea [14], coupled with sporadic outbreaks in several other countries [15–17], makes it a serious public health issue in areas of disease endemicity.

Despite the wide range of preventative approaches that have been attempted in the past 70 years, all have failed to develop an effective prophylactic vaccine [18]. Approaches have included the use of formalin-killed bacteria [19,20], inoculation with viable organisms followed by antimicrobial treatment [21], irradiated *Orientia tsutsugamushi* [22], subunit vaccines [23,24], and DNA vaccine [25]. Most of the vaccine trials resulted in short-term protection (generally less than one year), immunity to only the homologous strain, or no significant outcomes, especially in human infections. Immunity generated by the vaccine trials, or even after natural infections, does not last long and is poorly cross-reactive among numerous strains [7,18], thus reinfection with scrub typhus is relatively common in highly endemic areas [26]. To date, more than 20 strains have been reported, including the prototype strains Karp, Kato, and Gilliam [7]. Genetic analysis of the major outer membrane protein, the 56 kDa type-specific antigen (*tsa56*) unique to *O*. *tsutsugamushi*, revealed at least 11 definable genotype strains [7]. Although the TSA56 protein is an immunodominant antigen and has long been considered as a vaccine target, remarkable genetic heterogeneity among strains of *O*. *tsutsugamushi* limits cross-protective immunity against heterologous strains [18].

Selecting conserved antigens among different strains of *O*. *tsutsugamushi* is one of the critical issues to generating a clinically effective vaccine that produces cross-protective immunity against scrub typhus. Utilization of genome sequences obtained from bioinformatics through genomics and proteomics can expedite the vaccine discovery process by rapidly providing a set of potential candidates for vaccine antigen targets [27,28]. Previously, we reported the whole genome sequence of the *O*. *tsutsugamushi* Boryong strain [29] and profiled its global gene expression using a microarray system and proteomic approaches [30]. From our proteomic analysis, we predicted 10 outer membrane proteins unique to *O*. *tsutsugamushi*, two of which encode autotransporter proteins [30]. Analysis of two sequenced genomes, the *O*. *tsutsugamushi* Boryong and Ikeda strains [31], revealed four conserved genes (*scaA*, *C*, *D*, and *E*) encoding autotransporter proteins and *scaB* is duplicated in the Boryong strain but absent from the Ikeda strain [32]. Furthermore, genetic analysis using genomic DNAs from the three prototypes strains, Karp, Kato, and Gilliam, revealed that *scaA*, *C*, and *D* are present in all the strains tested but *scaB* and *scaE* are amplified differently in the different strains [33]. We also showed that specific antibody responses against ScaA and ScaC were observed in scrub typhus patients [33] and ScaC is involved in bacterial adhesion to eukaryotic host cells, potentially via interaction with host fibronectin [32]. Therefore, the conserved Sca proteins may play a role in bacterial pathogenesis and represent conserved targets for vaccine development. Recently, it was reported that other *Rickettsia* species, the sister clade of *Orientia*, also express multiple autotransporter proteins in their outer membrane and utilize them for the bacterial pathogenesis [34,35]. Not only that, an autotransporter protein, rickesstsial outer membrane protein B (rOmpB), was reported to elicit humoral immune responses that protect animals against lethal challenge [36].

In the current study, we examined the potential role of ScaA protein in *Orientia* pathogenesis and evaluate the protective attributes of Sca protein administration against lethal *O*. *tsutsugamushi* infection in mice. Our findings demonstrate that ScaA protein participates in bacterial adhesion. In addition, immunization with ScaA not only provides protective immunity against lethal challenge of the homologous strain, but also confers significant protection against heterologous strains when combined with TSA56. These results indicate that ScaA proteins could be a novel vaccine target for scrub typhus.

## Methods

### Ethics statement

Animal experiments were approved by the Seoul National University Hospital Institutional Animal Care and Use Committee (SNUH IACUC No.12–0331-C1A03) and performed in strict accordance with the recommendations in the National Guide Line for the care and use of laboratory animals. Ethical approval for this work was granted by the Institutional Review Boards of Seoul National University Hospital (IRB no. 0–1001–039–307).

### Cell culture

HeLa cells (ATCC CCL-2, American Type Culture Collection), L929 cells (ATCC NCTC929), Vero cells (ATCC CCL-81), and ECV304, an endothelial cell-like cell line [37], were maintained in DMEM (Welgene, Daegu, Korea) supplemented with 10% heat-inactivated fetal bovine serum (FBS) (Welgene), 100 U/mL penicillin and 100 μg/mL streptomycin (Gibco BRL) at 37°C in 5% CO_2_.

### Preparation of *O*. *tsutsugamushi*

The Boryong, Karp, and Kato strains of *O*. *tsutsugamushi* were purified using a modified Percoll gradient purification method [32,38]. *O*. *tsutsugamushi* was propagated in L929 cells. At 3 to 4 days post-infection, infectivity was determined using an indirect immunofluorescence assay (see below). When an infection rate of > 90% was achieved, the cells were harvested by centrifugation at 6000 × *g* for 20 min. The cell pellet was resuspended with Tris-sucrose (TS) buffer (33 mM Tris-Cl (pH 7.4) and 0.25 M sucrose) and homogenized using 100 strokes of a Polytron homogenizer (Wheaton Inc., Millville, NJ) followed by centrifugation at 200 × *g* for 5 min. The supernatant was then mixed with 40% Percoll (Pharmacia Fine Chemicals, Uppsala, Sweden) in TS buffer and centrifuged at 25,000 × *g* for 60 min. The bacterial band was collected and centrifuged at 77,000 × *g* for 30 min. The bacterial pellet was washed 3 times in TS buffer, resuspended in DMEM and stored in liquid nitrogen until use [39]. The infectivity titer of the inoculum was determined as previously described [40], with minor modifications. Infected-cell-counting units (icu) were calculated as follows: [(total number of cells used for infection) × (percentage of infected cells) × (dilution of the *O*. *tsutsugamushi* suspension)]/100. For infection assays, 1.0 × 10^7^ icu of *O*. *tsutsugamushi* were used to infect cells cultured in 6-well plates containing 1.0 × 10^6^ of host cells.

### Sequence analysis

Nucleotide sequences of *scaA* genes amplified from Gilliam, Karp, and Kato strains were deposited to GenBank under accession no. KM591910, KM591911, and KM591912, respectively. The *scaA* gene sequence from the Boryong strain was obtained from its genomic sequences (GenBank accession no. AM494475.1). Sequences of *tsa56* genes from each strain (AM494475.1 for Boryong, AY956315.1 for Gilliam, AY836148.1 for Karp, and GU120147.1 for Kato strain) were also used for comparative analysis. Nucleotide sequence alignments for constructing phylogenetic trees were processed by Clustal W with maximum likelihood method implemented in MEGA6 software [41]. The similarity and identity of those nucleotides and amino acids was calculated through Matrix Global Alignment Tool (MatGAT) version 2.03 [42]. The aligned nucleotide sequences were evaluated in SimPlot version 3.5.1 with Kimura (2-parameter) and Empiric Ts/Tv ratio settings [43]. The aligned amino acid sequences were analyzed through the BLOSUM62-referenced 100 amino acid sliding window analysis. The output values were calculated from R-Project (http://www.r-project.org/). Line graphs were visualized by GraphPad Prism software version (Graph-Pad Software Inc., La Jolla, CA). Repeat sequences within a *scaA* gene were also analyzed using Tandem Repeat Finder software [44].

### Cloning and expression of recombinant antigens

*scaA*, *scaB*, *scaC*, *scaE*, and *tsa56* were amplified from the genomic DNA of *O*. *tsutsugamushi* Boryong strain by PCR using the primer pairs listed in S1 Table. The PCR products were cloned into pET-28a or pGEX4T-1 vector (Novagen, Gibbstown, NJ). Full length *scaA* genes were also amplified from the genomes of Boryong, Gilliam, Karp, and Kato strains for sequence comparison. All constructs were sequenced to confirm in-frame cloning. Recombinant Sca and TSA56 proteins were purified from *E*.*coli* BL21 (DE3) harboring a recombinant plasmid encoding each bacterial protein. Following induction with isopropyl β-D-thiogalactoside (IPTG) (0.1 mM, Duchefa, Zwijndrecht, Netherlands) at 16°C for 16 h, the proteins were purified using Ni-nitrilotriacetic acid His-resin (Qiagen, Calrsbad, CA) or glutathione-Sepharose 4B columns (GE Healthcare, Piscataway, NJ) according to manufacturer’s instructions. The purified proteins were dialyzed against phosphate-buffered saline (PBS) in an Aside-A-Lyzer Dialysis Cassette (Therrmo scientific, Rockford, IL) at 4°C for overnight. After dialysis, purified proteins were treated with endotoxin removal columns (Thermo scientific) and endotoxin contamination was determined using the QCL-1000 kit (Lonza, Bloemfontein, South Africa) according to manufacturer’s instructions. All protein contained less than < 0.05 EU/mg of endotoxin.

### Antibodies and reagents

Both preimmune mouse serum and anti-Sca polyclonal mouse serum (produced from Balb/c mice immunized with purified Sca proteins; Cosmogenetech, Seoul, South Korea) were used for the experiments. Human sera were prepared from scrub typhus patients following institutional review board approval. Horseradish peroxidase (HRP)-conjugated anti-mouse or anti-human IgG secondary antibodies (Santa Cruz Biotech Inc., Santa Cruz, CA) were used for immunoblotting [32]. The Alexa Fluor 488- or Alexa Fluor 594-conjugated anti-mouse, and-human antibodies used in the immunofluorescence assays were purchased from Molecular Probes (Invitrogen). For the beadbinding assay, Fluoresbrite microparticles (1 μm; Polyscience Inc., Warrington, PA) containing rhodamine were conjugated to GST or GST-ScaA proteins by using a PolyLink protein coupling kit (Polyscience Inc.) in accordance with the manufacturer’s instructions.

### Bead-binding assay

HeLa cells (2.4 × 10^5^ cells in a 24-well plate) were incubated with Fluoresbrite microparticles (416 μg/well) conjugated to GST or GST-Sca proteins for 1 h, washed extensively with PBS, and fixed with 4% paraformaldehyde for 15 min [32]. Cells were subsequently incubated with ToPro-3 (Molecular Probes) for nuclear staining and observed under a confocal microscope or analyzed using a FACScan (Becton Dickinson).

### Cellular adhesion and invasion assays

Bacterial adhesion and invasion assays were performed as previously described [32]. Briefly, *E*. *coli* strains harboring a vector or pET28a encoding *scaA* gene were induced with IPTG and added to confluent monolayers of ECV304, HeLa, and Vero cells in serum-free media. Portions of the bacterium-containing media were plated to determine the number of CFU added to each host cell monolayer. Contact between bacteria and the mammalian cells was synchronized by centrifugation at 200 × *g*, and the preparations were incubated at 37°C for either 20 min or 60 min for the adherence and invasion assays, respectively. For the invasion assays, infected cells were washed extensively with PBS and incubated for 2 h with complete medium supplemented with 100 μg/ml of gentamicin to kill any extracellular bacteria [32]. For all *E*. *coli* assays, infected cells were washed extensively with PBS and the bacteria liberated by incubation with 0.1% Triton X-100 in sterile water. The lysate was then plated on LB agar to enumerate the cell-associated bacteria. The results were expressed as the percentages of bacteria recovered relative to the number of bacteria in the initial inoculum [32].

For antibody neutralization assays, HeLa cells or ECV304 cells were grown in a 24-well plate (2.4 × 10^5^ cells/well) and infected with *O*. *tsutsugamushi* or *E*. *coli* expressing ScaA in the presence of 1:100-diluted preimmune or anti-Sca polyclonal mouse serum. Association of *E*. *coli* with host cells were measured by CFU assays as mentioned above. To detect intracellular *O*. *tsutsugamushi*, infected cells were stained by differential immunofluorescence assay [38]. First, cells were washed three times with PBS, fixed with 4% paraformaldehyde, and incubated with an anti-TSA56 antibody, followed by Alexa Fluor 488-conjugated goat anti-mouse IgG to stain the cell-surface associated-bacteria. Next, cells were permeabilized in a 0.2% Triton X-100 solution for 15 min and incubated with scrub typhus patients’ sera for 1 h, followed by Alexa Fluor 633-conjugated goat anti-human IgG to stain intracellular bacteria. Cells were observed using an Olympus FV1000 laser confocal microscope (Olympus, Tokyo, Japan) and analyzed using the Fluoview software (Olympus).

### Immunofluorescence microscopy

Immunofluorescence microscopy was used to visualize *O*. *tsutsugamushi*. HeLa cells infected with *O*. *tsutsugamushi* were washed with PBS and fixed with 4% paraformaldehyde incubated with pooled scrub typhus human serum or anti-ScaA immune serum for 1 h, followed by incubation with Alexa Fluor 488-conjugated goat anti-mouse IgG and Alexa Fluor 594-conjugated mouse anti-rabbit IgG (Invitrogen) [32]. In some experiments, recombinant *E*. *coli* was stained with preimmune mouse serum, anti-ScaA serum, or anti-*E*. *coli* serum, followed by incubation with Alexa Fluor 488-conjugated mouse anti-rabbit IgG (Invitrogen) [32]. Cells were examined under an Olympus FV1000 laser scanning confocal microscope (Olympus). Images of cell sections were analyzed and processed using the Olympus Fluoview software (Olympus).

### ELISA

To determine the titer of antibodies specific to ScaA or TSA56 in the sera of immunized mice, immunoassay plates (96-well plates; Nunc, Rochester, NY) were coated with 100 μl of purified antigen at a concentration of 5μg/ml at 4°C overnight. The plates were then blocked for 2 h at room temperature with PBS containing 5% skim milk. 100 μl of serum samples serially diluted in 2-fold were incubated for 2 h at room temperature. After washing with PBS containing 0.05% Tween20 (PBST), horseradish peroxidase (HRP)-conjugated goat anti-mouse IgM, IgG_1_, or IgG_2c_ (Santa Cruz Biotechnology, Santa Cruz, CA) was added and incubated for 2h at room temperature. Wells were washed with PBST and incubated with 3,3′,5,5′-tetramethylbenzidine (TMB) peroxidase substrate solution (KPL, Gaithersburg, MD) for 10 min. The reactions were stopped by addition of 1M phosphoric acid solution. Absorbances were measured at 450 nm using a microplate reader (Beckman Coulter Inc., Fullerton, CA).

### Immunization of mice and challenges

For immunization experiments, 6- to 8-week-old female C57BL/6 mice (Orient Bio Inc., Seongnam, South Korea) were used. Groups (*n* = 5) of mice were immunized subcutaneously in the hind leg three times at two weeks interval. 20 μg of purified ScaA, ScaC, or TSA56 proteins in PBS emulsified 1:1 with 2% alhydrogel adjuvant (Invitrogen) was used for each immunization. Blood was collected from immunized mice after one week after each immunization to determine serum antibody titer. One week after the final immunization, mice were challenged intraperitoneally with 10 × or 100 × LD_50_ of *O*. *tsutsugamushi* strains. Body weight and mice survival was monitored for one month after bacterial challenge.

### Statistical analysis

The data was analyzed using the Graph Pad Prism 5.01 software. Statistical analysis of all the experimental data except survival rate was performed using the two-tailed Student’s t-test with 95% confidence interval. Data are expressed as the mean ± standard deviation. Statistical analysis on survival rates were performed using the Mantel-Cox Log Rank test. A *p*-value of < 0.05 was considered statistically significant.

## Results

### ScaA is expressed in *O*. *tsutsugamushi*

**Fig 1:**
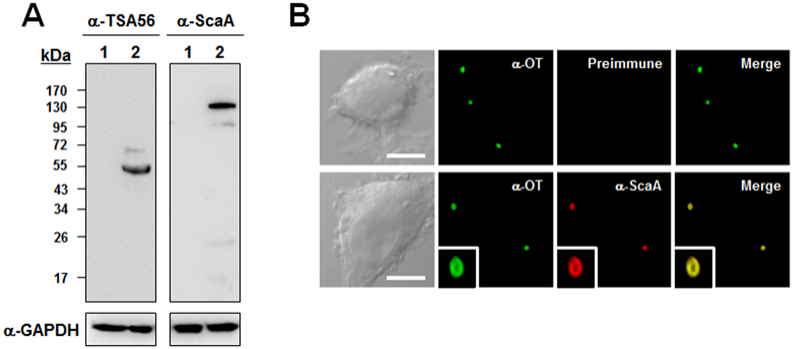
Expression of ScaA by *O*. *tsutsugamushi*. (A) Immunoblot analysis of whole proteins from L929 cells infected with *O*. *tsutsugamushi* proteins by using anti-ScaA serum (right panel). Anti-ScaA serum detected a protein with a molecular mass of approximately 150 kDa. Immunoblotting using anti-TAS56 was performed as a control (left panel). (B) Immunofluorescence confocal microscopy using preimmune serum or anti-ScaA serum (α-ScaA) showed ScaA in the *O*. *tsutsugamushi*-infected L929 cells. The left panels show bacteria stained with the pooled sera of scrub typhus patients (α-OT). Magnified images are shown in the lower panels (inset boxes). Scale bars, 5 μm.

In order to examine whether the ScaA is expressed in *O*. *tsutsugamushi*, we generated a polyclonal anti-ScaA antiserum by immunizing mice with purified ScaA passenger domain (amino acids 30 to 1000). The specificity of this antiserum was confirmed by ELISA and immunoblot analysis using the recombinant Sca proteins (S1 Fig). To identify endogenous ScaA protein in *O*. *tsutsugamushi*, the anti-ScaA serum was reacted with the cell lysates of *O*. *tsutsugamushi*-infected cells. Immunoblot analysis showed that a ~ 150 kDa protein was recognized by the anti-ScaA serum in infected cells but not in uninfected control (Fig. 1A). The full-length ScaA protein was predicted to have a mass of 156 kDa. Anti-ScaA serum was also weakly reacted with a few bands lower than ~ 150 kDa, suggesting a cross-reactive antigens or fragmented ScaA protein in infected cells. The TSA56 protein, a major outer membrane protein of *O*. *tsutsugamushi*, was used as a positive control [32]. To further confirm the specificity of the anti-ScaA antiserum for *O*. *tsutsugamushi*, intracellular bacteria were stained using the pooled sera of scrub typhus patients together with anti-ScaA serum or preimmune mouse serum. As shown in Fig. 1B, anti-ScaA serum readily detected the bacteria within the host cells, whereas the preimmune serum did not. In addition, we found that the ScaA proteins were located on the periphery of bacterial cells (Fig. 1B. lower panels, inset boxes). Taken together, these results confirm that the *scaA* gene is actively translated in *O*. *tsutsugamushi* within eukaryotic host cells and that the protein might be expressed on the outer membrane of the bacteria.

### ScaA mediates bacterial adhesion to host cells

**Fig 2:**
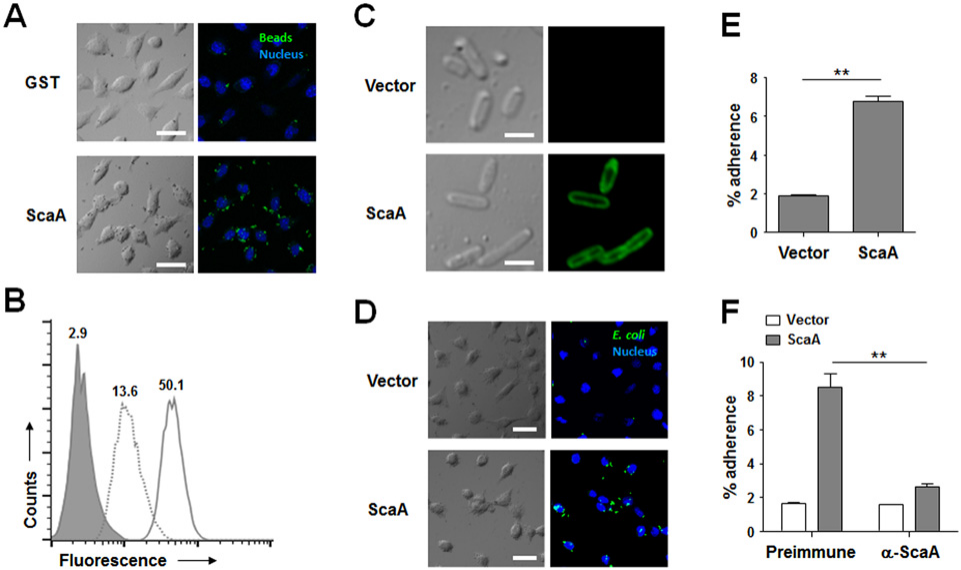
Adhesion function of ScaA. (A) HeLa cells were incubated with fluorescent microbeads coated with GST or GST-ScaA (ScaA) for 1 h, washed extensively, and fixed. Cell-bound microbeads (green) were visualized by fluorescence microscopy after staining of cell nuclei (blue). Scale bars, 10 μm. (B) Relative binding of the microbeads coated with GST (dotted line) or GST-ScaA (thick line) to HeLa cells was quantified directly using fluorescence-activated cell sorter (FACS) analysis. The gray histogram represents unbound cells (cells not incubated with microbeads). (C) Immunofluorescence microscopy using an anti-ScaA antibody revealed the presence of ScaA on the surface of the recombinant *E*. *coli* (lower panels). Preimmune serum did not detect the recombinant protein (upper panels). Scale bars, 5 μm. (D) *E*. *coli* transformed with the pET28a vector or with pScaA was induced with IPTG and incubated with HeLa cells. After being washed to remove adherent bacteria, the cells were fixed, permeabilized, and stained with an anti-*E*. *coli* antibody (green) and ToPro-3 for nuclear staining (blue). Scale bars, 10 μm. (E) CFU-based quantification of adherent *E*. *coli* transformed with the vector or pScaA was performed. The results are presented as percentages of adherent bacteria relative to the total bacterial input. Data are representative of three independent assays for each of the host cells. **, *p* < 0.01. (F) Inclusion of anti-ScaA serum in the medium (α-ScaA) significantly inhibited adhesion of *E*. *coli* expressing ScaA into host cells. After addition of anti-ScaA or preimmune serum into infection media, CFU-based quantification of adherent *E*. *coli* transformed with the vector or pScaA was performed. **, *p* < 0.01.

Recently, several studies reported that rickettsial Sca proteins mediate bacterial adherence to and/or invasion into mammalian host cells [32,45]. Therefore, we examined whether the *O*. *tsutsugamushi* ScaA protein could function as a virulence factor for bacterial adhesion and/or invasion. First, we performed a bead-binding assay using fluorescent microbeads (1 μm in diameter) covalently conjugated to either purified GST or GST-ScaA. Incubation of HeLa cells with GST-ScaA-conjugated beads resulted in marked binding to the host cells, even after extensive washing. The control beads linked to GST alone interacted only weakly with the HeLa cells (Fig. 2A) [32]. The interaction of the fluorescent beads with the host cells was quantified using flow cytometry (Fig. 2B). After fixation, the mean fluorescence intensity (MFI) of the HeLa cells incubated with beads conjugated to GST-ScaA dramatically increased (MFI = 50.1) compared to that of cells incubated with beads conjugated to GST (MFI = 13.6) or that of untreated cells [32].

To further verify the role of the *scaA* gene in bacterial adherence to host cells, we utilized a heterologous *E*. *coli* expression system [32]. The entire *O*. *tsutsugamushi scaA* open reading frame was cloned into the IPTG-inducible expression vector, pET-28a, to yield the plasmid pScaA as previously described [32]. ScaA was expressed in the *E*. *coli* strain BL21(DE3) and analyzed using anti-ScaA serum and confocal microscopy after fixation with 4% paraformaldehyde. As shown in Fig. 2C, ScaA was readily detectable on the surface of all the recombinant *E*. *coli* cells by anti-ScaA serum (lower panels) but not on bacteria harboring empty vector (upper panels). We next examined the ability of ScaA-expressing *E*. *coli* to adhere to monolayers of nonphagocytic host cells [32]. Epithelial (HeLa and Vero) and endothelial (ECV304) cells were incubated with recombinant *E*. *coli* harboring an empty vector or pScaA. The cells were then washed extensively to remove non-adherent bacteria, fixed, and analyzed under a confocal microscope after staining with an anti-*E*. *coli* antibody and the nuclear stain ToPro-3. Immunofluorescence analysis revealed that ScaA expression resulted in an increase in the number of adherent *E*. *coli* bacteria (Fig. 2D). This ScaA-mediated enhanced adhesion was verified by removing the adherent bacteria from the live host cells and counting them using a CFU-based quantification assay [32]. The assay confirmed that ScaA expression significantly increased bacterial adherence to HeLa cells (Fig. 2E) and other cell lines (S2A Fig). Therefore, the expression of *O*. *tsutsugamushi* ScaA on the outer surface of *E*. *coli* enhances bacterial adherence to nonphagocytic host cells. We also examined whether our anti-ScaA antibody could neutralize the adhesion of bacteria expressing ScaA to host cells. Treatment of the recombinant bacteria with the anti-ScaA antibody for 1 h before adding it to host cells reduced bacterial adhesion by approximately four fold (Fig. 2F) when compared to treatment with nonimmune serum, indicating that the anti-SacA antibody can block ScaA-mediated bacterial adhesion to host cells.

### ScaA vaccination provides protective immunity against *O*. *tsutsugmaushi* infection

**Fig 3:**
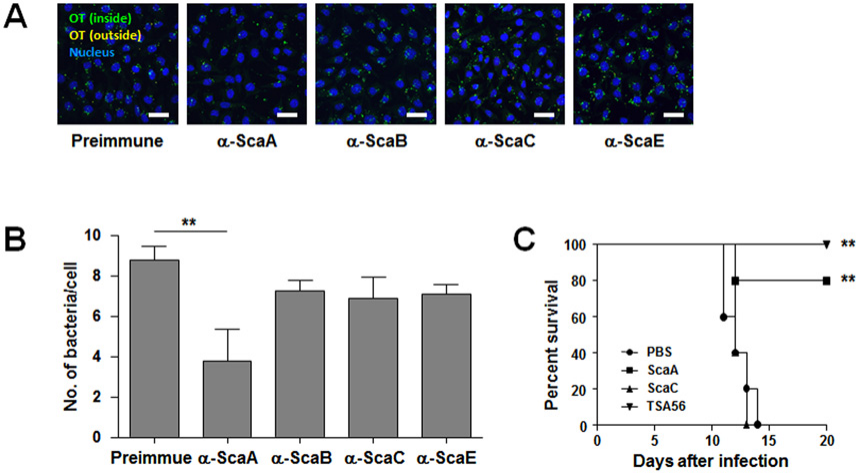
Protective role of anti-ScaA immunity. (A) Anti-ScaA antibody inhibited *O*. *tsutsugamushi* infection into host cells. HeLa cells were infected with the pathogen in the presence of the indicated anti-Sca antibodies or nonimmune serum. At 4 h after infection, bacterial infection was examined using confocal microscopy after differential immunoflourescent staining (see materials and methods). (B) The *O*. *tsutsugamushi* per host cell ratio was determined from three independent experiments in (A). **, *p* < 0.01. (C) Survival curves of immunized mice following lethal challenge with *O*. *tsutsugamushi*. Mice (*n* = 5/group) were immunized with the indicated antigen from the Boryong strain and challenged intraperitoneally with 100 x LD_50_ of *O*. *tsutsugamushi* Boryong strain. Their survival was monitored until all the surviving mice recovered from the disease. This graph is a representative survival curve of two experiments. **, *p* < 0.01 when compared with non-immunized group (PBS).

In order to confirm the neutralizing effect of anti-ScaA antibody on *O*. *tsutsugamushi* infection, HeLa cells were infected with the pathogen in the presence of various anti-Sca antibodies or nonimmune serum. At 4 h after infection, bacterial infection was examined by confocal microscopy after differential immunoflourescent staining and the *O*. *tsutsugamushi*/host cell ratio was determined (Fig. 3A and 3B). Presence of anti-ScaA antibody in the infection media significantly inhibited *O*. *tsutsugamushi* infection of host cells. The number of bacteria per cell was reduced by approximately 50% compared with the control group treated with nonimmune serum, whereas other anti-Sca antibodies failed to significantly inhibit bacterial infection, indicating that the anti-ScaA antibody could provide specific protective effect against bacterial infection.

This protective effect of the anti-ScaA antibody against *O*. *tsutsugamushi* infection was further investigated *in vivo* by challenging mice with 100 × LD_50_ of *O*. *tsutsugamushi* at 7 d after immunization. All the vaccine antigens were derived from the *O*. *tsutsugamushi* Boryong strain and mice were challenged with the same bacterial strain. As shown in Fig. 3C, a significant level of protection against the homologous strain was observed in the ScaA-immunized group as well as in the TSA56-immunized mice. In contrast, ScaC immunization did not provide any significant protection. Therefore, vaccination with a ScaA antigen could provide protective immunity against homologous strain infection as efficiently as TSA56, a dominant membrane antigen of *O*. *tsutsugamushi*.

**Fig 4:**
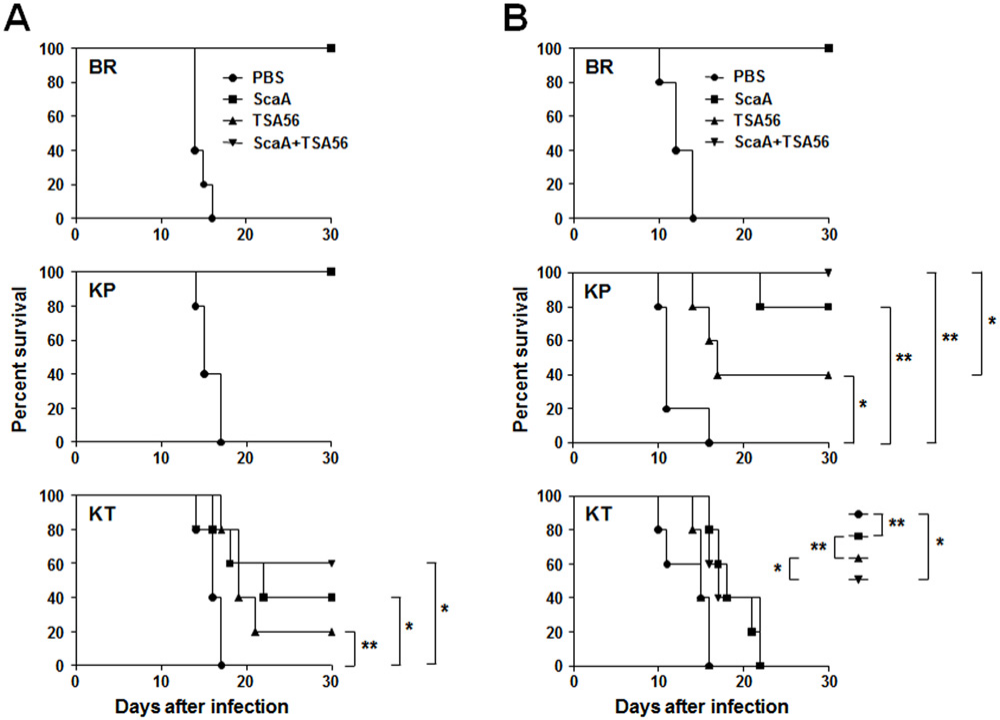
Protective role of ScaA or combined immunization against heterologous strain infection. Mice (*n* = 5/group) were immunized with the indicated antigens and challenged intraperitoneally with 10 x LD_50_ (A) or 100 x LD_50_ (B) of *O*. *tsutsugamushi*. Mice were immunized with antigens from the Boryong strain and challenged with the indicated strains (BR: Boryong, KP: Karp, KT: Kato). *p* value and median survival are summarized in S3 Fig. *, *p* < 0.05; **, *p* < 0.01.

Next, we tested whether the candidate bacterial antigens could provide protective immunity against heterologous strain infection. Each group of mice were immunized with the indicated antigens derived from the *O*. *tsutsugamushi* Boryong strain and then challenged with a low (10 × LD_50_) or high (100 × LD_50_) dose of Boryong, Karp, or Kato strains (Fig. 4 and S3 Fig). We confirmed significant increases of both type 1 (IgG_2C_) [46] and type 2 (IgG_1_) antibodies against ScaA and/or TSA56 after third immunization (S4 Fig). Following infection with *O*. *tsutsugamushi*, mock-immunized mice began to lose body weight between 8–12 d after inoculation, depending on the bacterial doses and strains challenged, and lost 10–25% of body weight before they expired (S5 Fig). All the unimmunized mice had expired by 10–17 d after infection. The immunized mice survived after infection with *O*. *tsutsugamushi* maintained normal body weight during the experiment, but the expired ones rapidly lost their body weight from 4–8 d before death. All the mice immunized with ScaA or TSA56 were protected from the homologous Boryong strain regardless of infection dose, and were also protected against low dose (10 × LD_50_) Karp strain infection. When mice were challenged with high dose (100 × LD_50_) Karp strain infection, all the mice immunized with both ScaA and TSA56 survived and 80% of ScaA-immunized mice were protected. Although TSA56 immunization also provide significant protection (40% survival, *p* value = 0.017) compared to mock-immunized control group, the protection level was significantly (*p* value = 0.049) lower than that afforded by vaccination with both ScaA and TSA56 antigens. In the groups challenged with low dose Kato strain, groups immunized with both ScaA and TSA56 showed the best protective effect (60% survival) and TSA56 immunization provided only 20% survival. Immunization with ScaA also provided significant protection (40% survival). Although the level of protection afforded by ScaA (median survival = 22 d) was higher than that of TSA56 (median survival = 19 d), the difference was not statistically significant (*p* > 0.05). In contrast, ScaA vaccination (median survival = 18 d) significantly prolonged the survival of mice compared to TSA56 and mock-immunization (*p* < 0.01, median survival = 15 d in both groups) when mice were challenged with high dose of Kato strain. Immunization of TSA56 together with ScaA provided a similar level of protection as that observed in the ScaA immunization group even though all the challenged mice ultimately succumbed to pathogen infection.

## Discussion

Despite continuous efforts to develop a vaccine for scrub typhus since World War II, an effective vaccine is not yet available. Earlier human studies using inactivated whole bacteria failed to show evidence of protection [19,20]. A more recent study using a formalin-inactivated antigen prepared from chicken egg-adapted *O*. *tsutsugamushi* showed protection in mice against the same strains used for immunization, but failed to protect from infections with other strains [47]. The requirement of a biosafety level 3 facility for the cultivation of the pathogen is an additional barrier for the mass production of a cost-effective vaccine using the whole bacterial antigen. Therefore, whole cell vaccine products may not be practical and economically feasible, and the majority of the recent studies of potential scrub typhus vaccines mainly focus on selecting subunit antigens as vaccine candidates [18,48]. Before the genomic era, most of the vaccine studies were performed using antigens recognized by sera obtained from immunized animals and infected humans, such as 22-. 47-. 56-. 58-, and 110 kDa proteins [18,48]. Among them, the type-specific antigen (TSA), a 56 kDa protein, has long been tested as a vaccine candidate since it is highly immunogenic and plays an important role in *O*. *tsutsugamushi* attachment to and invasion into host cells [38,49,50]. Thus far, TSA56 has been the best antigen to provide protective immunity in mouse infection models, but only to homologous strain infection due to its antigenic diversity [23]. Another conserved major antigen, a 47 kDa protein, has been tested as a vaccine antigen [51]. However, this bacterial antigen failed to provide significant protection against homologous strain challenge and did not improve vaccine efficacy even when combined with TSA56 [51]. In addition, the 47 kDa antigen may induce cross-reactivity against human serine proteases due to sequence homology, and thus potentially contributes to autoimmune responses or enhanced pathology in some scrub typhus patients [52]. Immunogenic 22- and 110-kDa proteins have also been considered as vaccine antigens, but their efficacy have never been proven in *in vivo* infection models [48].

Based on the genomic information of two *O*. *tsutsugamushi* strains [29,31], there are 161 *O*. *tsutsugamushi*-specific genes that are absent in all other sequenced *Rickettsia* species [31]. Among them, we identified 58 genes that are present in both the Boryong and Ikeda strains but are absent in all other bacteria species in the NCBI database (cutoff e-value < 10^-20^) (S2 Table). In the whole bacterial proteome analysis, 17 *O*. *tsutsugamushi*-specific genes including *tsa56* and *scaA* were identified to be translated [30]. Here, we showed that ScaA, an autotransporter protein of *O*. *tsutsugamushi*, is expressed on the bacterial periphery and functions as a bacterial adhesion factor (Fig. 1 and 2). Autotransporter proteins of gram-negative bacteria share a common sequence organization: a signal peptide followed by an N-terminal passenger domain and a C-terminal translocator domain [53]. The sequences and functions of the passenger domains can be quite diverse and are frequently associated with various virulent phenotypes, including bacterial adhesion, invasion, biofilm formation, and cytotoxicity [53]. The apparent role of autotransporter proteins in virulence and host cell interactions naturally make them potential targets for the design of novel vaccines directed against human pathogens [32,54]. For example, a major virulence factor of *Bordetella pertussis*, pertactin that mediates bacterial adhesion to the lung epithelium [55] and resistance to neutrophil-mediated clearance [56], has been successfully used to provide the acellular components of a pertussis vaccine [57]. The passenger domain of the *Haemophilus influenzae* autotransporter protein, Hap, which mediates attachment and entry into epithelial cells as well as attachment to extracellular matrix proteins [58], elicits significant antibody responses and protects preimmunized mice from nasopharyngeal colonization [59].

**Fig 5:**
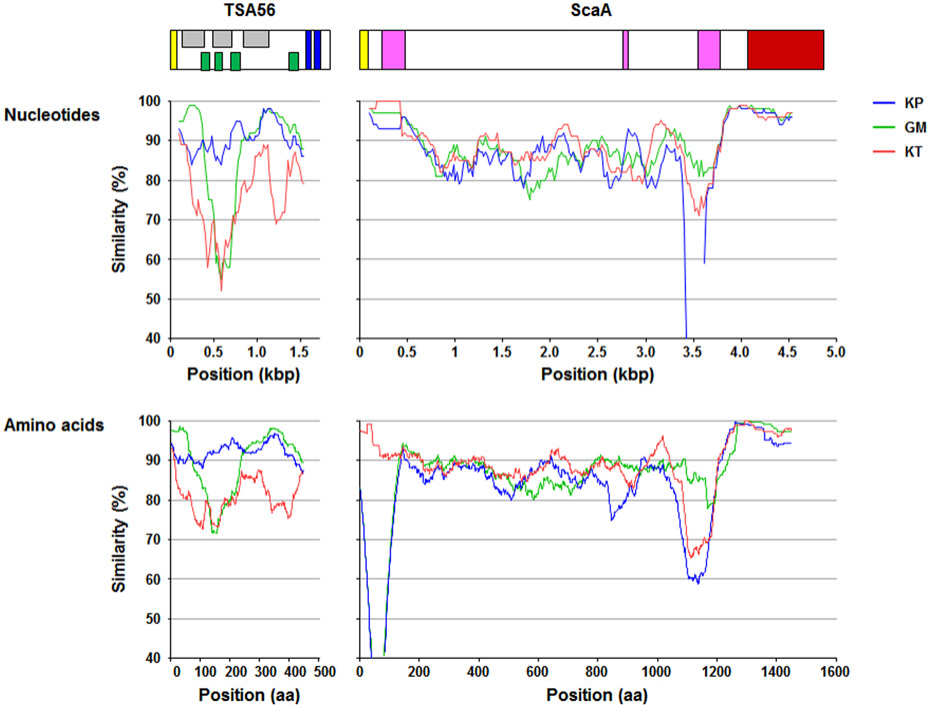
Similarity plots of a set of *tsa56* and *scaA* sequences from the indicated strains compared to sequences from the Boryong strain. Each plotted point is the percent identity within a sliding window of 100 bp or 100 amino acids wide centered on the position plotted, with a step size between points of 10 bp or amino acids. Diagrams above the graphs show the relative sizes of TSA56 and ScaA proteins and their sequence motiffs. Yellow box: signal peptide, gray box: antigenic domain, green box: variable domain, blue box: transmembrane domain, pink box: repeated sequences, brown box: autotransporter domain.

In this study, we examined the neutralizing activity of antibodies against four Sca proteins (ScaA, B, C, and E) encoded in the *O*. *tsutsugamushi* genome and found that only the antibody against ScaA inhibited bacterial infection in a cell culture model, whereas antibodies against other Sca proteins of *O*. *tsutsugamushi* had marginal effects (Fig. 3). In addition, immunization with ScaA provided protective immunity against *O*. *tsutsugamushi* infection in mice as efficiently as TSA56, whereas ScaC failed to induce protection, indicating that ScaA could provide specific and protective immunity against *O*. *tsutsugamushi*, at least against the homologous strain (Fig. 3C). When combined with TSA56, ScaA immunization significantly enhanced protective immunity against infection with heterologous strains, resulting in better survival or extended half-life of infected mice (Fig. 4). To our knowledge, this is the most promising result of scrub typhus vaccination against infection of heterologous strains in a mouse model. When we compared the sequences of *scaA* from the four different strains, the overall level of sequence similarity of *scaA* nucleotides (83.5 ~ 87.5%) and amino acids (81.0 ~ 88.4%) is similar to those of *tsa56* (nucleotides: 77.5 ~ 88.4%, amino acids: 78.8 ~ 90.2%) (S6 Fig). However, a similarity plot shows that *tsa56* has more local variation among the four strains than *scaA* (Fig. 5). Sequence variation observed in *scaA* is mainly due to the differential presence of repeated sequences found in the 5’-region (nucleotides 203 ~ 241 in Boryong strain) and 3’-end of the passenger domain (nucleotides 3243 ~ 3314 in Boryong strain) of each strain. When we examined the neutralizing activity of anti-ScaA antibody generated by immunizing ScaA protein from Boryong strain, it showed less inhibitory effect on the cellular invasion of Kato strain than Boryong strain (S2B Fig), suggesting that the sequence variation of ScaA may also affect *in vitro* neutralizing activity of anti-ScaA antibody. It remains to be determined whether the variable regions and their repeated sequences affect the antigenicity or neutralizing activity of antibodies against ScaA protein. Since protective immunity against *O*. *tsutsugamushi* infection is provided by antigen-specific IFN-γ-producing T cells [60,61] as well as humoral immunity [23,62], the protective role of ScaA-specific Th1 cells also needs to be investigated. Nevertheless, the passenger domains of ScaA proteins from different strains are relatively well conserved and those conserved areas could make it a better antigen for scrub typhus vaccine for targeting multiple strains of *O*. *tsutsugamushi*.

Recently, several infection models using mice have been proposed to study pathologic changes and vaccine development for scrub typhus [63–66]. Intradermal or intravenous inoculation of the pathogen partially represented the specific pathology of human scrub typhus [64,65]. An infection model using *O*. *tsutsugamushi*-infected mites to mimic the natural transmission was also shown that the species of infected chigger and their *O*. *tsutsugamushi* genotypes produced different clinical presentations in ICR mice [63]. Previously, diverse strains of mice showed differential morbidity and mortality to the infection with specific strains of *O*. *tsutsugamushi* [67,68]. Therefore, various factors such as infection routes and genetic backgrounds of host and the pathogen may affect the susceptibility and disease severity of scrub typhus. In the current study, we used C57BL/6 inbred mice model after intraperitoneal injection of *O*. *tsutsugamushi* strains, which resulted in 100% mortality when unimmunized. Valid models in C57BL/6 mice also open the opportunity to study genes involved in the mechanisms of immunity and pathogenesis by the use of gene knockout mice [69]. The development of animal models that accurately portray human scrub typhus is an important step toward understanding and managing disease [65,66]. Although there are differences in target cells of *O*. *tsutsugamushi* infection and the disease progression depending on the route of infection and the genotypes, these models closely parallels the clinical course and pathological legions described from lethal scrub typhus in human and, therefore, may provide valuable tools to characterize the molecular and cellular factors responsible for immunological pathogenesis of scrub typhus [65]. Further studies on the bacterial virulence mechanisms [39,70,71] and the underlying mechanisms of immunological pathogenesis in human scrub typhus patients [72] should also be followed to improve our understanding for the weak and transient immunity against the bacterial infection in human and to facilitate the development of effective vaccine for scrub typhus.

## Supporting Information

S1 Table Primer sequences used in this study. (DOCX)

S2 Table *O*. *tsutsugamushi*-specific genes shared by the Boryong and Ikeda strains. (DOCX)

S1 Fig Antibody responses against Sca antigens in immunized mice. Sera from mice immunized with the indicated antigen were diluted 1:100 and used for ELISAs. Data are presented from triplicate assays.
(DOCX)

S2 Fig Effect of ScaA and anti-ScaA antibody on bacterial adhesion and invasion. (A) CFU-based quantification of adherent *E*. *coli* transformed with the vector or pScaA was performed. The results are presented as percentages of adherent bacteria relative to the total bacterial input. Data are representative of three independent assays for each of the host cells. (B) Anti-ScaA antibody generated by immunization with ScaA antigen from Boryong strain differentially inhibited *O*. *tsutsugamushi* Boryong (BR) or Kato (KT) strain infection into host cells. ECV304 cells were infected with the indicated strain in the presence of anti-ScaA antibody or nonimmune serum. At 4 h after infection, bacterial infection was examined using confocal microscopy after differential immunofluorescent staining and the *O*. *tsutsugamushi* per host cell ratio was determined from three independent experiments.
(DOCX)

S3 Fig Statistical analysis on survival rates. Statistical analysis on survival rates were performed using the Mantel-Cox Log Rank test. A *p*-value of < 0.05 was considered statistically significant (red). *:undefined
(DOCX)

S4 Fig Antibody responses of different isotypes in immunized mice. Antibody titers in mice (*n* = 3) at one week after third immunization presented in relative units (RU) as serial dilution of serum relative to antibody end-point titers. *: titer < 100
(DOCX)

S5 Fig Body weight change of mice challenged with diverse *O*. *tsutsugamushi* strains. Mice (n = 5/group) were immunized with the indicated antigens and challenged intraperitoneally with 10 x LD_50_ (A) or 100 x LD_50_ (B) of *O*. *tsutsugamushi* (the same sets in Fig. 4). Mice monitored and weighed daily for a month after inoculation of the pathogen and the average body weight of the surviving mice of each group is presented.
(DOCX)

S6 Fig Identity and similarity of *tsa56* and *scaA* sequences among different strains. Nucleotides and amino acids sequences from the indicated strains of *O*. *tsutsugamushi* were compared. Nucleotide sequence alignments for constructing phylogenetic trees were processed by Clustal W with the maximum likelihood method. The similarity and identity of those nucleotides and amino acids were calculated through Matrix Global Alignment Tool (MatGAT) (see Methods section). BR: Boryong, KP: Karp, GM: Gilliam, KT: Kato.
(DOCX)
