# Supplementary material for: Immunization with an Autotransporter Protein of Orientia tsutsugamushi Provides Protective Immunity against Scrub Typhus
Source: PLoS Negl Trop Dis. 2015 Mar 13;9(3):e0003585. doi: 10.1371/journal.pntd.0003585 (PMC4359152; doi:10.1371/journal.pntd.0003585)
Supplement: S1 Table — (DOCX) [file pntd.0003585.s001.docx]

S1 Table. Primer sequences used in this study


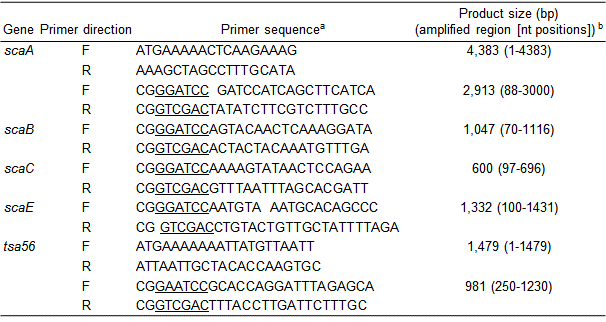


^a^ Restriction enzyme sites are underlined.

^b^ From genes of the Boryong strain.
