# Supplementary material for: Immunization with an Autotransporter Protein of Orientia tsutsugamushi Provides Protective Immunity against Scrub Typhus
Source: PLoS Negl Trop Dis. 2015 Mar 13;9(3):e0003585. doi: 10.1371/journal.pntd.0003585 (PMC4359152; doi:10.1371/journal.pntd.0003585)
Supplement: S2 Table — (DOCX) [file pntd.0003585.s002.docx]

S2 Table. *O. tsutsugamushi*-specific genes shared by the Boryong and Ikeda strains^a^


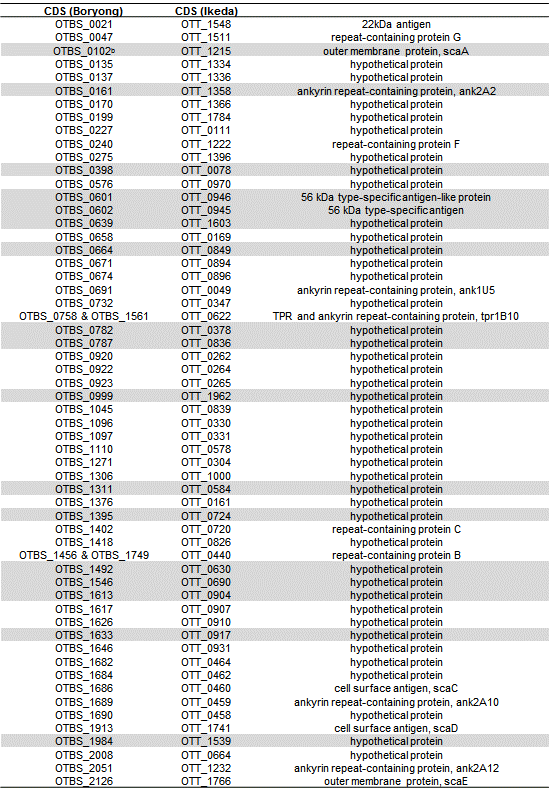


^a^ *O. tstusgamushi*-specific genes shared by the two strains were reported in a previous study [[1](#_ENREF_1)].

^b^ Genes with a gray background were detected in proteomic analysis in a previous study [[2](#_ENREF_2)].

**References**

1. Nakayama K, Yamashita A, Kurokawa K, et al. The Whole-genome sequencing of the obligate intracellular bacterium Orientia tsutsugamushi revealed massive gene amplification during reductive genome evolution. DNA Res **2008**; 15:185-99.

2. Cho BA, Cho NH, Min CK, et al. Global gene expression profile of Orientia tsutsugamushi. Proteomics **2010**; 10:1699-715.
