# Supplementary material for: Immunization with an Autotransporter Protein of Orientia tsutsugamushi Provides Protective Immunity against Scrub Typhus
Source: PLoS Negl Trop Dis. 2015 Mar 13;9(3):e0003585. doi: 10.1371/journal.pntd.0003585 (PMC4359152; doi:10.1371/journal.pntd.0003585)
Supplement: S1 Fig — Sera from mice immunized with the indicated antigen were diluted 1:100 and used for ELISAs. Data are presented from triplicate assays. (DOCX) [file pntd.0003585.s003.docx]

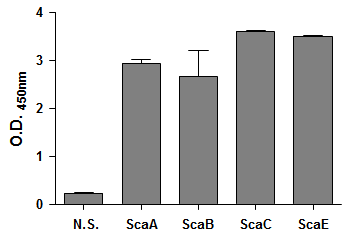


**S1 Fig.** Antibody responses against Sca antigens in immunized mice. Sera from mice immunized with the indicated antigen were diluted 1:100 and used for IgG ELISAs. Data are presented from triplicate assays.
