# Supplementary material for: Immunization with an Autotransporter Protein of Orientia tsutsugamushi Provides Protective Immunity against Scrub Typhus
Source: PLoS Negl Trop Dis. 2015 Mar 13;9(3):e0003585. doi: 10.1371/journal.pntd.0003585 (PMC4359152; doi:10.1371/journal.pntd.0003585)
Supplement: S2 Fig — (A) CFU-based quantification of adherent E. coli transformed with the vector or pScaA was performed. The results are presented as percentages of adherent bacteria relative to the total bacterial input. Data are representative of three independent assays for each of the host cells. (B) Anti-ScaA antibody generated by immunization with ScaA antigen from Boryong strain differentially inhibited O. tsutsugamushi Boryong (BR) or Kato (KT) strain infection into host cells. ECV304 cells were infected with the indicated strain in the presence of anti-ScaA antibody or nonimmune serum. At 4 h after infection, bacterial infection was examined using confocal microscopy after differential immunofluorescent staining and the O. tsutsugamushi per host cell ratio was determined from three independent experiments. (DOCX) [file pntd.0003585.s004.docx]

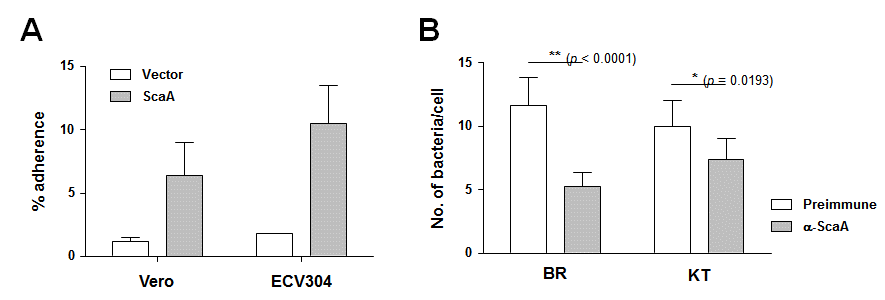


**S2 Fig.** Effect of ScaA and anti-ScaA antibody on bacterial adhesion and invasion. (A) CFU-based quantification of adherent *E. coli* transformed with the vector or pScaA was performed. The results are presented as percentages of adherent bacteria relative to the total bacterial input. Data are representative of three independent assays for each of the host cells. (B) Anti-ScaA antibody generated by immunization with ScaA antigen from Boryong strain differentially inhibited *O. tsutsugamushi* Boryong (BR) or Kato (KT) strain infection into host cells. ECV304 cells were infected with the indicated strain in the presence of anti-ScaA antibody or nonimmune serum. At 4 h after infection, bacterial infection was examined using confocal microscopy after differential immunoflourescent staining and the *O. tsutsugamushi* per host cell ratio was determined from three independent experiments.
