# Supplementary material for: Immunization with an Autotransporter Protein of Orientia tsutsugamushi Provides Protective Immunity against Scrub Typhus
Source: PLoS Negl Trop Dis. 2015 Mar 13;9(3):e0003585. doi: 10.1371/journal.pntd.0003585 (PMC4359152; doi:10.1371/journal.pntd.0003585)
Supplement: S3 Fig — Statistical analysis on survival rates were performed using the Mantel-Cox Log Rank test. A p-value of < 0.05 was considered statistically significant (red). *:undefined (DOCX) [file pntd.0003585.s005.docx]

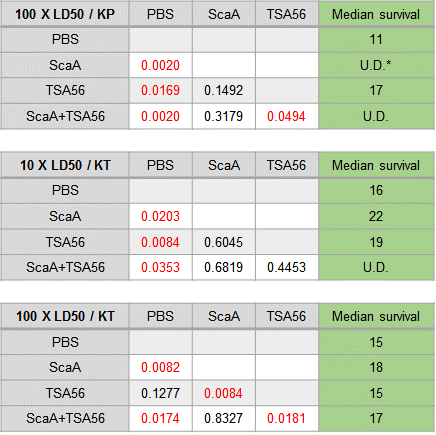


**S3 Fig.** Statistical analysis on survival rates were performed using the Mantel-Cox Log Rank test. A *p*-value of < 0.05 was considered statistically significant (red). *:undefined
