# Supplementary material for: Immunization with an Autotransporter Protein of Orientia tsutsugamushi Provides Protective Immunity against Scrub Typhus
Source: PLoS Negl Trop Dis. 2015 Mar 13;9(3):e0003585. doi: 10.1371/journal.pntd.0003585 (PMC4359152; doi:10.1371/journal.pntd.0003585)
Supplement: S4 Fig — Antibody titers in mice (n = 3) at one week after third immunization presented in relative units (RU) as serial dilution of serum relative to antibody end-point titers. *: titer < 100 (DOCX) [file pntd.0003585.s006.docx]

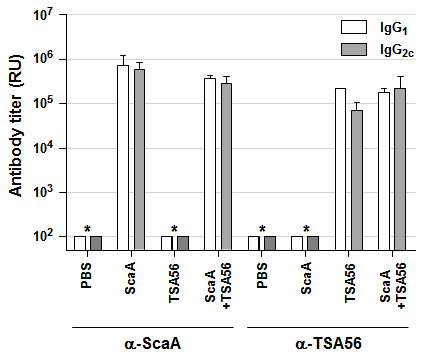


**S4 Fig.** Antibody responses of different isotypes in immunized mice. Antibody titers in mice (*n* = 3) at one week after third immunization presented in relative units (RU) as serial dilution of serum relative to antibody end-point titers. *: titer < 100
