# Supplementary material for: Immunization with an Autotransporter Protein of Orientia tsutsugamushi Provides Protective Immunity against Scrub Typhus
Source: PLoS Negl Trop Dis. 2015 Mar 13;9(3):e0003585. doi: 10.1371/journal.pntd.0003585 (PMC4359152; doi:10.1371/journal.pntd.0003585)
Supplement: S5 Fig — Mice (n = 5/group) were immunized with the indicated antigens and challenged intraperitoneally with 10 x LD50 (A) or 100 x LD50 (B) of O. tsutsugamushi (the same sets in Fig. 4). Mice monitored and weighed daily for a month after inoculation of the pathogen and the average body weight of the surviving mice of each group is presented. (DOCX) [file pntd.0003585.s007.docx]

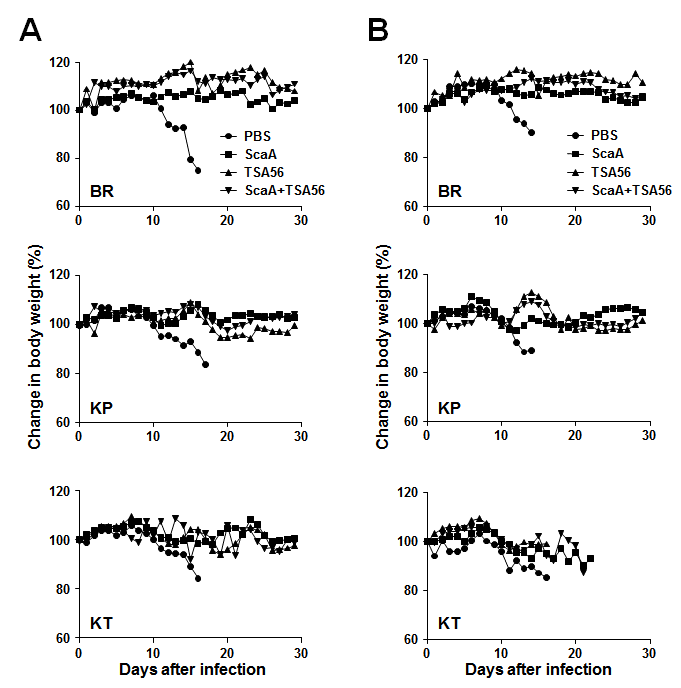


**S5 Fig.** Body weight change of mice challenged with diverse *O. tsutsugamushi* strains. Mice (n = 5/group) were immunized with the indicated antigens and challenged intraperitoneally with 10 x LD_50_ (A) or 100 x LD_50_ (B) of *O. tsutsugamushi* (the same sets in Figure 4). Mice monitored and weighed daily for a month after inoculation of the pathogen and the average body weight of the surviving mice of each group is presented.
