# Supplementary material for: Immunization with an Autotransporter Protein of Orientia tsutsugamushi Provides Protective Immunity against Scrub Typhus
Source: PLoS Negl Trop Dis. 2015 Mar 13;9(3):e0003585. doi: 10.1371/journal.pntd.0003585 (PMC4359152; doi:10.1371/journal.pntd.0003585)
Supplement: S6 Fig — Nucleotides and amino acids sequences from the indicated strains of O. tsutsugamushi were compared. Nucleotide sequence alignments for constructing phylogenetic trees were processed by Clustal W with the maximum likelihood method. The similarity and identity of those nucleotides and amino acids were calculated through Matrix Global Alignment Tool (MatGAT) (see Methods section). BR: Boryong, KP: Karp, GM: Gilliam, KT: Kato. (DOCX) [file pntd.0003585.s008.docx]

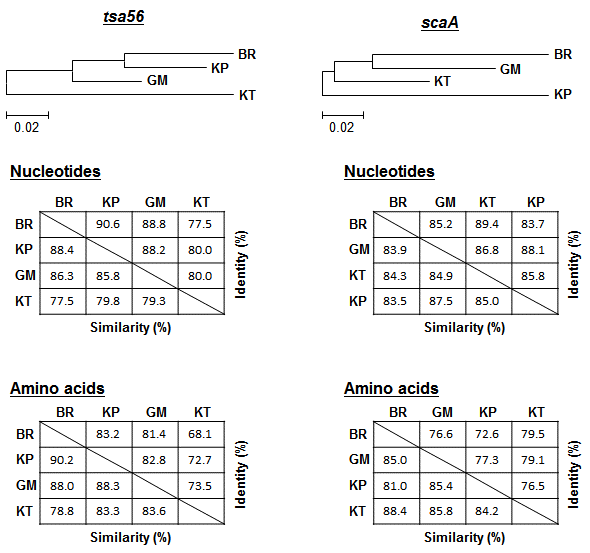


**S6 Fig.** Nucleotides and amino acids sequences from the indicated strains of *O. tsutsugamushi* were compared. Nucleotide sequence alignments for constructing phylogenetic trees were processed by Clustal W with the maximum likelihood method. The similarity and identity of those nucleotides and amino acids were calculated through Matrix Global Alignment Tool (MatGAT) (see Methods section). BR: Boryong, KP: Karp, GM: Gilliam, KT: Kato.
